# An automated, web-based triage tool may optimise referral pathways in elective orthopaedic surgery: A proof-of-concept study

**DOI:** 10.1177/20552076231152177

**Published:** 2023-01-26

**Authors:** Alexandra L. Stanley, Thomas C. Edwards, Martin D. Jaere, Johnathan R. Lex, Gareth G. Jones

**Affiliations:** 1 Faculty of Medicine, 4615 Imperial College London, London, UK; 2 MSk Lab, 4615 Imperial College London, London, UK; 3 Division of Orthopaedic Surgery, Department of Surgery, 7938 University of Toronto, Toronto, Canada

**Keywords:** Orthopaedic, surgery, knee, e-triage, digital health, medical informatics, chatbot, screening, computer-assisted diagnosis

## Abstract

**Introduction:**

Knee pain is caused by various pathologies, making evaluation in primary-care challenging. Subsequently, an over-reliance on imaging, such as radiographs and MRI exists. Electronic-triage tools represent an innovative solution to this problem. The aims of this study were to establish the magnitude of unnecessary knee imaging prior to orthopaedic surgeon referral, and ascertain whether an e-triage tool outperforms existing clinical pathways to recommend correct imaging.

**Methods:**

Patients ≥18 years presenting with knee pain treated with arthroscopy or arthroplasty at a single academic hospital between 2015 and 2020 were retrospectively identified. The timing and appropriateness of imaging were assessed according to national guidelines, and classified as ‘necessary’, ‘unnecessary’ or ‘required MRI’. Based on an eDelphi consensus study, a symptom-based e-triage tool was developed and piloted to preliminarily diagnose five common knee pathologies and suggest appropriate imaging.

**Results:**

1462 patients were identified. 17.2% (*n* = 132) of arthroplasty patients received an ‘unnecessary MRI’, 27.6% (*n* = 192) of arthroscopy patients did not have a ‘necessary MRI’, requiring follow-up. Forty-one patients trialled the e-triage pilot (mean age: 58.4 years, 58.5% female). Preliminary diagnoses were available for 33 patients. The e-triage tool correctly identified three of the four knee pathologies (one pathology did not present). 79.2% (*n* = 19) of participants would use the tool again.

**Conclusion:**

A substantial number of knee pain patients receive incorrect imaging, incurring delays and unnecessary costs. A symptom-based e-triage tool was developed, with promising performance and user feedback. With refinement using larger datasets, this tool has the potential to improve wait-times, referral quality and reduce cost.

## Introduction

Knee pain affects approximately one quarter of the general population and is a common presenting complaint to primary and secondary care physicians.^1,2^ Unsurprisingly this constitutes a significant socioeconomic burden to the healthcare system, with the management of knee osteoarthritis (KOA) alone costing billions of pounds globally each year. This figure is predicted to grow due to an ageing and increasingly overweight population.^3–5^ Not only is KOA costly to manage, but evidence shows it to severely limit activity, ability to work and ability to manage other comorbid conditions, such as diabetes and hypertension.^6^

Knee pain can be secondary to a variety of pathologies, and the subtleties of presenting symptoms and clinical findings can make an accurate evaluation challenging for primary care physicians (PCPs).^7^ Understandably, this leads to uncertainty regarding the appropriate choice and timing of imaging, with an over reliance on advanced imaging, such as magnetic resonance imaging (MRI), to arrive at a diagnosis.^7^ Whilst MRI provides a detailed assessment of the soft tissue structures of the knee, it is an expensive and overburdened resource and there is little evidence to support the large volumes of PCP-requested MRIs each year.^8,9^ Indeed, the use of MRI as a primary screening tool is deemed inappropriate and for KOA it has poor sensitivity, with plain radiographs preferred instead.^8–15^ It is widely accepted that, for KOA, radiographs should be utilised before other imaging modalities, given their easy accessibility, relatively low cost and ability to detect bony pathology.^16^

Efforts such as ‘The Choosing Wisely Campaign’ have attempted to reduce unnecessary over-investigation and treatment of patients, however, their impact on improving clinical practice has been limited.^17,18^ A more novel approach, which has made great advancements since the COVID-19 pandemic, is the development of electronic triage (e-triage) tools.^19,20^ A variety of orthopaedic e-triage tools, capable of pre-hospital triage and diagnosis, are now in use. However, so far only one has been designed to assist in diagnosing knee pathology using a patient's history, and was found to generate multiple possible diagnoses for each patient, with low specificity.^21^ Whilst most tools focus on the initial triaging of patients and determining potential diagnoses, none have been developed to determine a patient's subsequent imaging needs. We therefore propose the development of an e-triage tool to be used in primary care that is capable of predicting a patient's most likely diagnosis and imaging requirements, prior to their initial secondary care appointment with an orthopaedic specialist. If effective, the e-triage tool has the potential to improve patient throughput, reduce wait-times, minimise the number of wasted appointments and unnecessary MRIs, thus reducing overall costs.

The aims of this study were (1) retrospectively establish the magnitude of unnecessary imaging in knee pain patients at a single academic hospital and (2) prospectively develop and ascertain whether an electronic chatbot triage system might be capable of outperforming existing clinical pathways with regards to the investigation of knee pain prior to arrival in secondary care.

## Methods

### Current state

#### Setting

We retrospectively identified patients presenting between January 2015 and December 2020 at a single academic hospital in London. The inclusion criteria were all patients over the age of 18 who were newly referred to an orthopaedic surgery knee clinic by a PCP and underwent any knee arthroplasty or arthroscopy procedure. Duplicates were collated under one entry and patients with incomplete records, such as those with missing clinic or referral letters were excluded. Patients who had, at any time, previously seen an orthopaedic surgeon for the same pathology in the same knee, and those referred for revision surgery were excluded. To reduce the heterogeneity of diagnoses and associated lack of guidelines, patients treated conservatively during this period were also excluded. The study received prospective institutional approval (Imperial College Healthcare Trust reference: TRA_166).

#### Data collection

Data was obtained using electronic clinic letters and imaging records. Collected variables included; patient demographics (such as age and sex), diagnosis and surgery received, referral source, imaging type (if any), timing and appropriateness of imaging. Timing was categorised as; ‘prior to’, ‘on the day of’ or ‘after’ the initial secondary care appointment. ‘Prior to’ was defined as imaging performed before the initial orthopaedic surgeon consultation, up to 6 months before the referral date. Appropriateness of imaging (defined below) was categorised as ‘correct’, ‘unnecessary MRI’ or ‘required MRI’.

#### Outcome definitions

We defined appropriateness based on the ‘British Association for the Surgery of the Knee’ (BASK) guidelines, which state that plain radiographs should be the first investigation when OA is suspected, but if soft tissue injury is suspected, MRI is the first line investigation.^12^ An ‘unnecessary MRI’ was defined as an MRI prior to secondary care referral in a patient who subsequently underwent knee arthroplasty for KOA. MRIs requested by the secondary care provider were not included, given that these were used to facilitate surgical planning or 3D patient specific guides rather than diagnosis. Patients who underwent arthroscopic soft-tissue surgery (e.g. for meniscal tear (MT) or ACL repair) and for whom an MRI scan had to be requested and organised at their first secondary care appointment were categorised as ‘required MRI’. Patients who had plain radiographs prior to knee arthroplasty for KOA, or an MRI prior to an arthroscopic soft-tissue procedure were considered to have had the ‘correct’ imaging.

### e-Triage tool proof-of-concept

#### Software development

An e-triage tool (chatbot) was developed using the *Amazon LEXbot* platform (Amazon, Washington, USA). The chatbot questions were derived from the results of an eDelphi consensus by a panel of 17 International knee ‘experts’ (consultant academic knee arthroplasty and sports surgeons), which identified key symptoms or patient factors for five common knee pathologies ((1) acute non-degenerative meniscal tear (MT); (2) knee osteoarthritis (KOA); (3) anterior cruciate ligament (ACL) injury; (4) posterior cruciate ligament (PCL) injury; (5) patellofemoral disease (PFD)). The eDelphi consisted of three rounds of questions related to knee symptoms, with consensus on each item considered when over 70% of experts agreed. The chatbot questions were phrased in plain English with ‘yes’ or ‘no’ answers required (Appendix 1). This question structure aimed to minimise the effects of individual patients’ competencies at describing their pain and symptoms, whilst also ensuring answers were answered as objectively as possible. Question branching was applied, with subsequent questions depending on previous responses. For example, if a patient indicated a traumatic onset to their knee problems, subsequent questions would further assess the injury, however, if there was no traumatic history, these questions were excluded.

#### Pilot study

A pilot study of the chatbot was carried out in a single knee clinic at the same central London academic hospital. This clinic constituted 101 new referrals, all of whom were contacted to participate in the study. Informed consent was given verbally by 77 patients who were willing to participate. The link to the chatbot triage tool was emailed to consenting participants prior to their appointment. Participants completed the chatbot on the e-triage tool website. Upon completion of the chatbot, a link to a questionnaire collecting data on satisfaction of chatbot use was made available to participants. This questionnaire assessed ease of use, ease of understanding, whether they would use the chatbot again, and an open text field for additional comments. A five-point Likert scale assessing whether patients strongly agreed, agreed, disagreed, strongly disagreed or remained neutral for each of the statements was used.

The ground truth for the preliminary diagnosis was arrived at by a consensus between two or more consultant knee surgeons. These surgeons were present at the clinic and had access to available imaging, patient notes and examination findings from the initial consultation visit. For each condition, the proportion of reported symptoms related to that diagnosis as identified by the eDelphi, was calculated. The diagnosis with the greatest proportion of reported symptoms was the suggested diagnosis recommended by the e-triage tool. This diagnosis was compared to the ground truth diagnosis. The e-triage tool then suggested appropriate imaging, based on its suspected diagnosis. As per BASK guidelines, if OA was suspected then plain-film radiographs were recommended, whilst if soft tissue injury (MT, ACL, PCL, PFD) was suspected the e-triage tool recommended MRI.^12^ The overall accuracy of the e-triage tool's imaging suggestion was calculated.

## Results

### Current state

A total of 1462 patients met the inclusion criteria for this study. There were 649 (44.4%) males and 813 (55.6%) females, with a mean age of 60.0 years (standard deviation (SD): 15.2, range: 19.3–92.3). Of these, 767 patients (52.5%) underwent knee arthroplasty, and 695 patients (47.5%) knee arthroscopy.

Seventy percent (*n* = 537) of the arthroplasty group and 77.6% (*n* = 539) of the arthroscopy group had received the ‘correct’ imaging prior to their first appointment. Seventeen percent (*n* = 132) of the arthroplasty group had received an ‘unnecessary MRI’ prior to their first clinic appointment.

**Figure 1.**
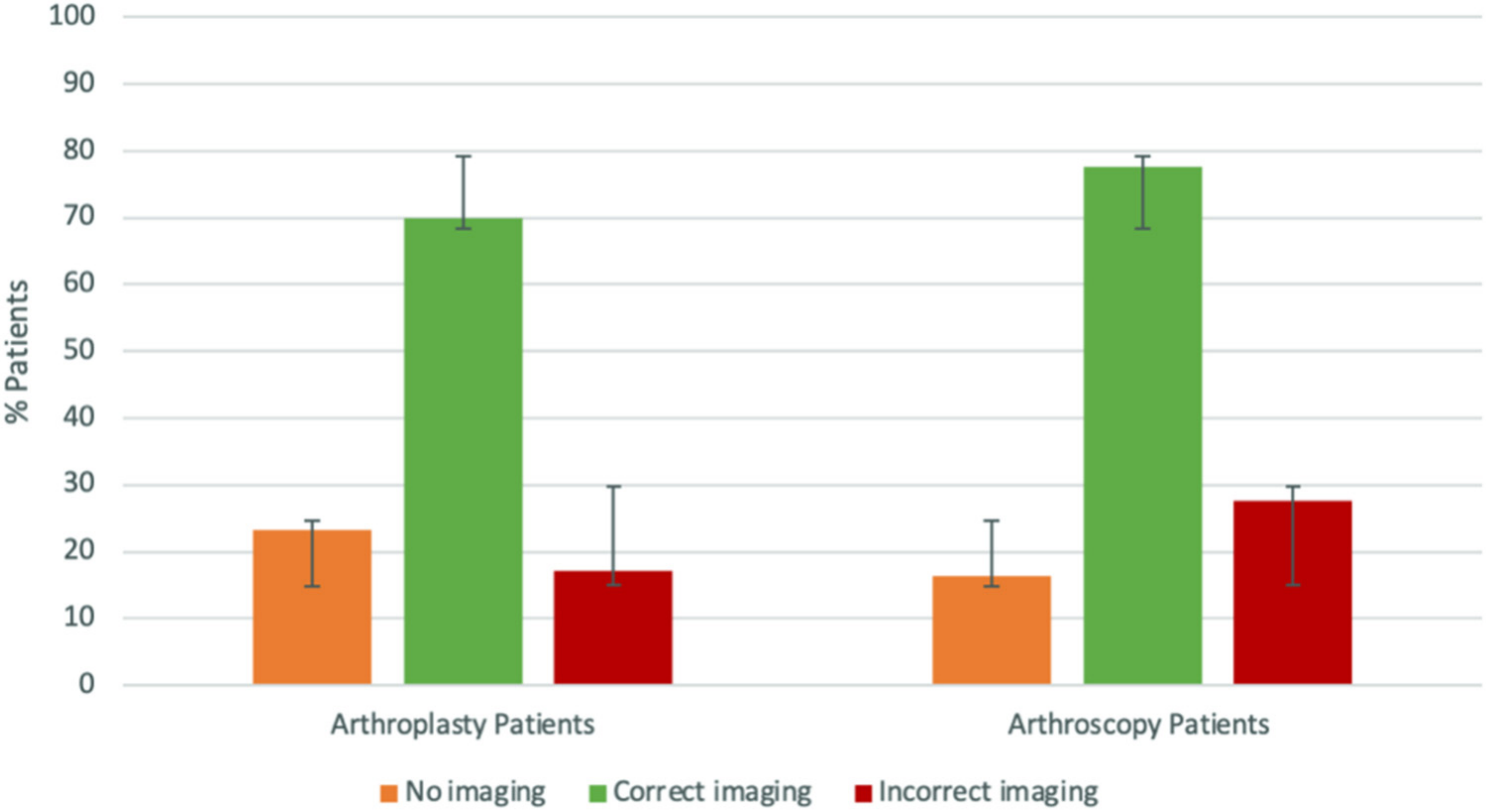
The imaging received by patients prior to their initial clinic appointment.

Twenty percent of patients (*n* = 292) arrived without any prior imaging (*n* = 179 arthroplasty and 113 arthroscopy). Amongst patients undergoing arthroscopy, 27.6% (*n* = 192) ‘required MRI’ and an ensuing follow-up appointment (Figure 1 and Table 1). Of the arthroscopy patients that required an MRI with follow-up appointment, this resulted in a mean delay of 3.1 months (SD: 2.6, range: 0.2–20.19) between their initial appointment and follow-up post-MRI. In the arthroplasty group, 204 (26.6%) required a radiograph on the day of their first clinic appointment.

− In the arthroplasty patients (*n* = 767); correct imaging = plain radiograph prior to appointment, incorrect imaging = magnetic resonance imaging (MRI) prior to appointment. − In the arthroscopy patients (*n* = 695); correct imaging = MRI prior to appointment, incorrect imaging = no/incorrect imaging before appointment therefore requiring MRI for review at follow-up appointment. N.B. bars do not equate to 100%, as some patients may have received both ‘correct’ and ‘incorrect’ imaging.

**Table 1.** Imaging status of patients.

		Arthroplasty		Arthroscopy	
		*n*	%	*n*	%
Total *n*		767	100.0	695	100.0
Referred by	GP	517	67.2	562	80.9
	ESP	247	32.1	130	18.7
	MSK Dr	3	0.4	3	0.4
No imaging performed prior to initial appointment		179	23.3	113	16.3
Imaging before appointment	XR	537^a^	70.0^a^	152	21.9
	MRI	132^b^	17.2^b^	539^a^	77.6^a^
Imaging on day of appointment	XR	204	26.6	152	21.9
	MRI	1	0.1	0	–
Imaging ordered for FU	XR	18	2.3	19	2.7
	MRI	111	14.5	192^c^	27.6^c^

The imaging status prior to initial clinic appointment of patients undergoing arthroplasty and arthroscopy between January 2015 and December 2020.

^a^ Correct imaging performed.

^b^ Unnecessary imaging performed (unnecessary MRI).

^c^ Incorrect/no imaging performed, so had to be referred for further imaging with follow-up appointment (superfluous appointment). N.B. some patients received a follow-up MRI despite having undergone imaging prior to their appointment due to the requirement for more up to date imaging.

*Abbreviations:* GP = general practitioner; ESP = extended scope practitioner; MSK Dr = GP with special interest in musculoskeletal conditions; XR = plain-film radiograph; MRI = magnetic resonance imaging; FU = follow-up.

### e-Triage model pilot study

Seventy-seven patients consented to be sent the e-triage tool. The e-triage tool assessed a total of 60 symptoms and patient factors. Fifteen items (25.0%) were considered ‘MT symptoms’, 19 (31.7%) ‘KOA symptoms’, 13 (21.7%) ‘ACL symptoms’, nine (15.0%) ‘PCL symptoms’ and 15 (25.0%) ‘PFD symptoms’. Thirteen (21.7%) of the symptoms assessed were indicated for more than one pathology.

A total of 41 (53.2%) participants completed the e-triage tool; 17 (41.5%) male and 24 (58.5%) female with a mean age of 58.4 years (SD 17.2, range 18–85). Preliminary diagnoses were available for 33 (80.5%) of the participants. Two (5.9%) patients had more than one possible preliminary diagnosis. Nineteen participants (57.6%) had a preliminary diagnosis of KOA, nine participants (27.3%) had a MT, three (9.1%) an ACL injury, and three (9.1%) PFD. No patients attending the knee clinic were suspected of having a PCL injury.

**Figure 2.**
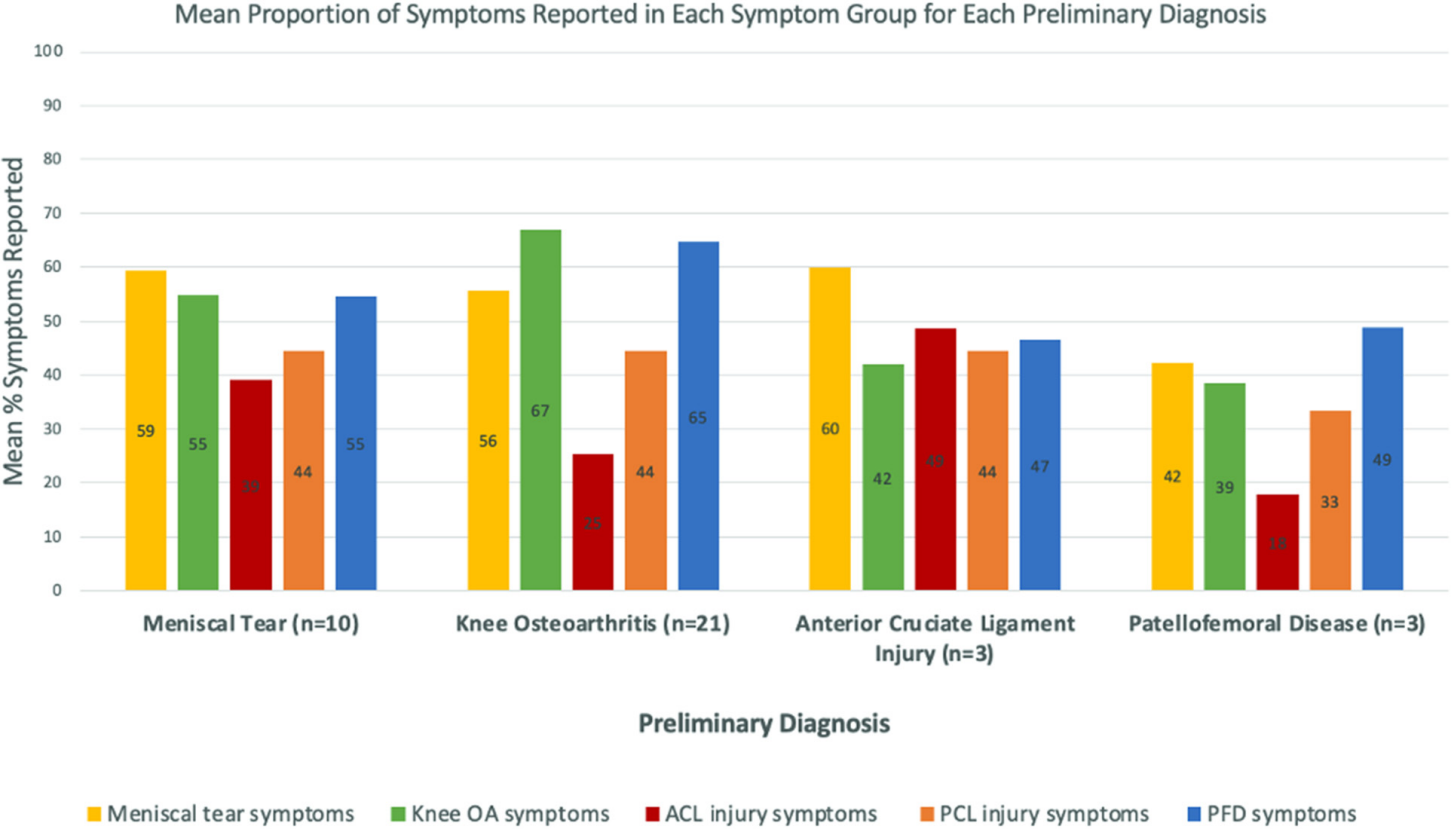
Graph showing the mean proportion of reported symptoms in each symptom group for each preliminary diagnosis. *n* = number of patients in each provisional diagnosis domain. *Abbreviations:* OA = osteoarthritis; ACL = anterior cruciate ligament; PCL = posterior cruciate ligament; PFD = patellofemoral disease.

A provisional diagnosis of ACL injury (*n* = 3) was the only pathology group that did not have the highest proportion of reported symptoms in the ‘correct’ symptom group. On average, ACL patients reported a higher proportion of MT symptoms (mean 60.0%, SD: 30.6%, range: 33.3%–93.3%) than ACL symptoms (ACL symptoms: mean 48.7%, SD: 29.1%, range: 15.4%–69.2%). Other pathologies (MT, KOA, PFD) had the highest proportion of reported symptoms in the correct corresponding symptom group (Figure 2 and Table 2). Overall, based on the e-triage tool's suspected diagnosis, the e-triage tool recommended the correct imaging in 69.7% (*n* = 23) of cases.

**Table 2.** Mean proportion of symptoms reported in each symptom group for each pathology.

Provisional diagnosis	Symptom group	*n*	Mean %	Standard deviation	95% CI lower bound	95% CI upper bound
**Acute non-degenerative meniscal tear**	**MT**	10	59.3^a^	18.2	47.3	68.7
	**KOA**	10	54.7	12.7	46.8	62.1
	**ACL**	10	39.2	24.4	25.4	53.8
	**PCL**	10	45.6	23.1	33.3	61.1
	**PFD**	10	54.6	17.2	44.0	64.7
**Knee osteoarthritis**	**MT**	21	55.6	16.8	48.6	62.2
	**KOA**	21	66.9^a^	13.8	61.2	72.4
	**ACL**	21	25.3	15.0	19.0	31.9
	**PCL**	21	44.4	17.2	27.0	51.9
	**PFD**	21	64.8	13.4	59.0	70.2
**ACL injury**	**MT**	3	60.0^a^	30.6	33.3	93.3
	**KOA**	3	42.1	10.5	31.6	52.6
	**ACL**	3	48.7	29.1	15.4	69.2
	**PCL**	3	44.4	11.1	33.3	55.6
	**PFD**	3	46.7	24.0	20.0	66.7
**Patellofemoral disease**	**MT**	3	42.7	25.2	13.3	60.0
	**KOA**	3	38.6	16.9	26.3	57.9
	**ACL**	3	17.9	17.8	7.7	38.5
	**PCL**	3	33.3	19.2	11.1	44.4
	**PFD**	3	48.9^a^	20.4	10.0	66.7

Chatbot outcomes were assessed to calculate the mean number of reported symptoms for each symptom group and these were compared to the patient's preliminary clinic diagnosis.

^a^ Indicates highest proportion of reported symptoms.

*Abbreviations:* CI = confidence interval; MT = acute non-degenerative meniscal tear; KOA = knee osteoarthritis; ACL = anterior cruciate ligament; PCL = posterior cruciate ligament; PFD = patellofemoral disease.

### Patient satisfaction

**Figure 3.**
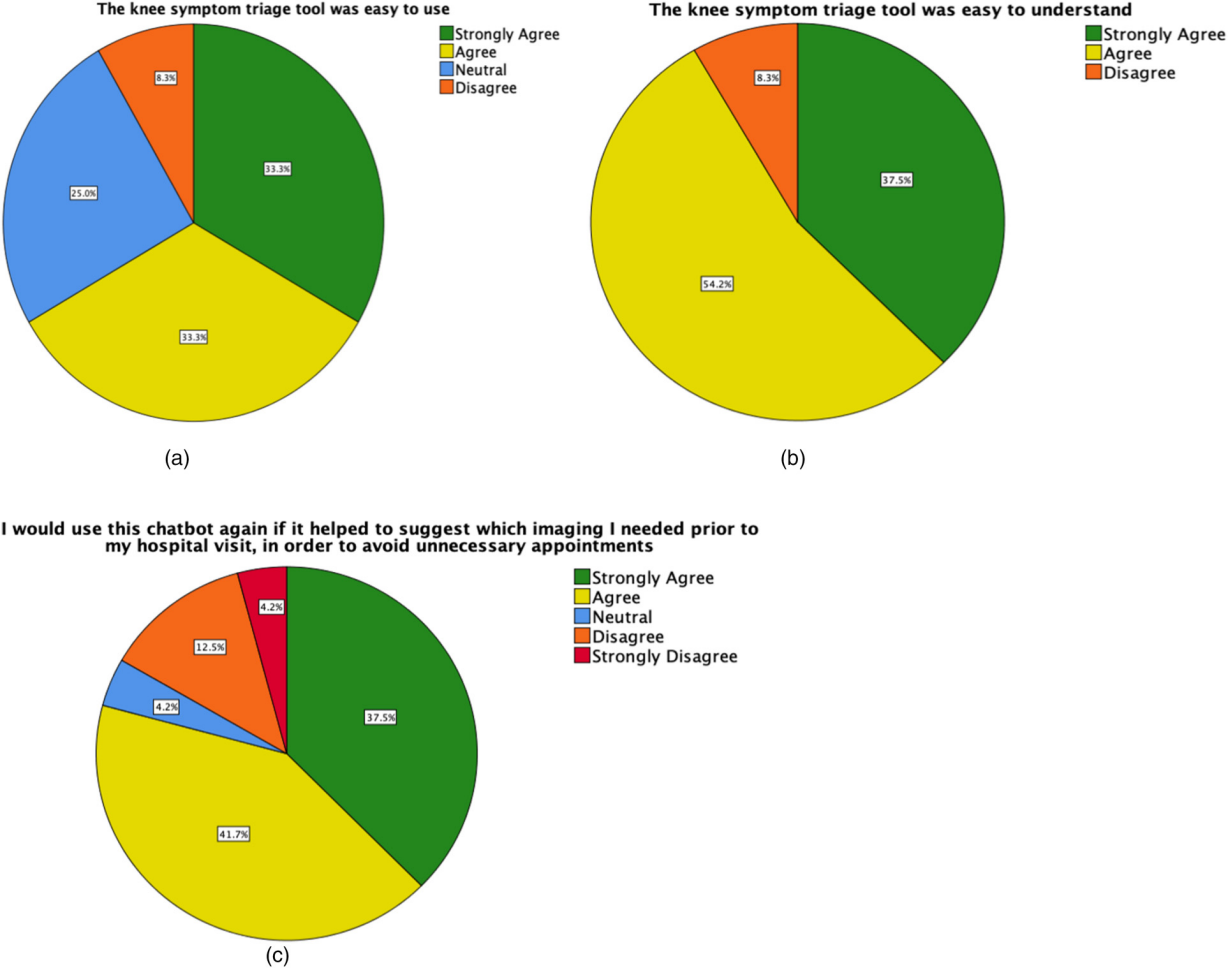
Patient responses to the e-triage tool satisfaction questionnaire. Upon completion of the e-triage tool, patients were asked whether; (a) The e-triage tool was easy to use. (b) The e-triage tool was easy to understand. (c) They would use the e-triage tool again.

Twenty-four (58.5%) participants completed the satisfaction questionnaire. The majority of patients either agreed or strongly agreed that the e-triage tool was easy to use and easy to understand. Only two (8.3%) participants felt the e-triage tool was hard to use and difficult to understand. When given the prompt ‘I would use this e-triage tool again if it helped to identify which imaging I required prior to my hospital visit, in order to avoid unnecessary appointments’, 19 (79.2%) participants either ‘strongly agreed’ or ‘agreed’ with the statement. One (4.2%) participant remained neutral and four (16.7%) ‘disagreed’ or ‘strongly disagreed’ (Figure 3).

Participants acknowledged that the yes/no question design kept the e-triage tool simple, however, several participants suggested that a ‘sometimes’ or scale option should be available, as symptoms were often intermittent and variable. It was also noted that the opportunity to give further explanation of their symptoms should be offered at an earlier stage in the questioning process, rather than at the end.

## Discussion

Our retrospective review of patients referred with knee pain to a single National Health Service (NHS) trust between January 2015 and December 2020 highlighted a problem with the pre-hospital work-up of new referrals. Notably, one in five (20.0%) patients, managed with arthroplasty or arthroscopy, presented to clinic having received no prior imaging. In contrast, approximately one in six (17%) of all patients received an unnecessary MRI, and over one in four (27%) patients required an MRI and subsequent follow-up appointment. A pilot study of an e-triage chatbot tool to address this issue produced promising results, with the chatbot tool able to identify three of the four pathologies assessed, by identifying the highest proportion of reported symptoms for each pathology group. Moreover, 79.2% of patients stated they would use the e-triage tool again. This suggests that with further refinement an e-triage tool has the potential to assist primary care in determining the correct pre-hospital investigation.

Whilst our study analyses the use of pre-hospital MRIs in patients referred for a specialist orthopaedic opinion, a number of other studies have assessed the appropriateness of primary care MRI referrals in general. They suggest that our findings might under-represent this, with a recent, large retrospective review finding that only 33% of patients with a PCP requested MRI went on to see an orthopaedic specialist.^8,10,22^ This is supported by data demonstrating that MRIs requested by PCPs have been shown to have a significantly higher rate of ‘normal’ outcomes when compared to those requested by secondary care specialists, with one study concluding that ‘inappropriate investigations’ are three times more likely to have a ‘normal’ result.^8,10,22^

Inadequate information at the time of consultation decreases patient satisfaction and increases the risk of chronic knee complaints and disability.^23–26^ It also has financial implications, and by applying standard NHS tariffs (£110 for an MRI and £163 for an outpatient appointment) to our results for 1400 patients with knee pain, we calculate that £14,520 was wasted on ‘unnecessary MRIs’ and £31,296 on ‘superfluous clinic appointments’. Extrapolating our results to the 312,316 primary knee replacements carried out across the UK between January 2017 and December 2019 would equate to almost £2 million wasted on unnecessary MRIs alone.^27^ Furthermore, this may be an underestimate given that a large study quoted the true cost of an MRI to be £295 per patient.^11^

Other studies have explored the use of chat-based triage tools with similarly encouraging results.^28–30^ Lai et al. highlight how the use of artificial intelligence (AI) and e-triage has the ability to enhance patient care; by relieving the strain on an overburdened system, both patient access and healthcare capacity can be improved.^30^ This is even more pertinent today, given the effect COVID-19 has had on telemedicine and the switch to remote consultations. In 2018, Quoro, a chat-based interface, was developed for triaging medical patients.^28^ Natural language processing algorithms were employed to acquire basic information, extract patient symptoms and suggest a diagnosis.^28^ Testing was carried out on 30 scenarios, and showed an overall mean precision of 0.82.^28^ Although these results are encouraging, testing was performed using patient vignettes which are unlikely to reflect the complexity of real-life patients.

Despite promising advancements in e-triage tools, their ability to give accurate diagnoses is variable and its real-world application still faces hurdles.^20,31,32^ Encouragingly, all diagnoses apart from ACL injury, would have been correctly identified by our e-triage tool, by simply identifying which symptom group had the highest proportion of reported answers. The e-triage tool's misdiagnosis of ACL injury may be unsurprising, as studies have shown ligamentous injury to represent a diagnostic predicament for PCPs.^7^ The patients with suspected ACL injury, had the highest proportion of reported symptoms in the MT group. As the aim of our developed triage tool is to recommend appropriate investigations, this slightly mitigates the error, as both conditions require the same diagnostic imaging (MRI), so the clinical suggestion recommended by the e-triage tool's algorithm would not be affected.^8^ Additionally, it would not be unsurprising if these patients were subsequently found to also have a MT, as is the case in up to 63% of ACL injuries.^33^ Lastly, this pilot study only captured three patients with ACL tears, therefore additional data is required to make more accurate assumptions.

With regards to suggesting appropriate imaging, the e-triage tool demonstrated a similar accuracy to the current standard of care. However, the e-triage tool would have the potential benefit of improved efficiency by automating the whole process and more importantly, its accuracy should improve with the collection of more data and utilisation of more sophisticated data analysis techniques, such as machine learning.

Additional concerns with the implementation of e-triage tools in healthcare include issues surrounding privacy and hesitancy around the use of AI from patients.^19,34^ However, studies have found that although some patients questioned the quality, trustworthiness and accuracy of e-triage tools, many were receptive to future use, as they were deemed useful and time-saving tools.^19^ This is coherent with the results of our e-triage tool satisfaction questionnaire, where the majority of users stated they would be happy to use the tool again, thus suggesting positive future implications. Similar user satisfaction questionnaires have been shown as useful in assessing usability and aiding the development of new applications.^35^

Not only must patients’ concerns be taken into account, but those of the PCPs must also be considered, especially given our desire for our chatbot to be used in the primary-care setting. Whilst we did not directly assess PCPs attitudes towards our e-triage tool, other studies have examined their attitude towards AI and telemedicine more generally.^36,37^ One study highlights some existential anxiety from PCPs towards AI and also feeling threated by AI technology, however, another study found PCP's largely have positive intentions to use telemedicine and e-triage tools in the future.^36^ Whilst this reluctance could pose some difficulties in implementing our chatbot tool, Pikkemaat et al. suggest that empowering PCP's self-efficacy towards the use of digital tools is key.^37^

There are several limitations to this study. Although we had a relatively large sample size for our retrospective audit, we only assessed patients who underwent arthroplasty or arthroscopy, thus failing to consider those who underwent a different procedure or form of treatment and those patients who underwent imaging at the request of their PCP, but never received review by an orthopaedic specialist. Therefore, we are likely to have significantly underestimated the size of the problem. Additionally, we cannot discount the possibility that some imaging may have been wrongly classed as ‘inappropriate’, as we cannot know which pathology the PCP was suspecting initially. Despite this, we deem it unlikely that initial suspicions varied significantly from ultimate diagnosis and treatment, due to each pathology's relatively distinct symptomology.

Although initial testing of our e-triage tool demonstrated positive results, it was only on a relatively small sample. We received 41 responses, despite 77 patients agreeing to complete the e-triage tool. This might suggest that patients had difficulties accessing or answering the e-triage tool, possibly due to lack of technological ability in the elderly population. However, every patient we spoke to had access to an email address and/or access to a close family member or friend who could assist them in completing the e-triage tool. Lastly, having only three patients in each of the ACL and PFD group and no patients suspected of PCL injury prevents us inferring any accuracy with regards to these diagnoses.

### Further work

Whilst our initial pilot results are largely positive, further work needs to be done to ensure the chatbot can have a positive impact on patient care, whilst also easing some of the strain on PCPs and the medical imaging service. Most notably, collection of a much larger dataset is vital. Once obtained, a machine learning algorithm will be developed and applied to our e-triage tool to attempt to improve the accuracy. Following this refinement, the e-triage tool requires assessment through a randomised control trial involving primary-care services to establish whether a tangible improvement in pre-hospital knee imaging and efficiency is seen.

## Conclusion

We found that a substantial proportion of knee pain patients do not receive the correct imaging prior to their initial outpatient appointment, resulting in inefficient patient flow and increased costs to the NHS. Preliminary testing of our e-triage tool, developed with the aim of addressing this issue, shows that by assessing key symptoms, prediction of a patient's pathology and thus the imaging they require is feasible. Initial outcomes of the e-triage tool and user feedback are promising and indicate the potential use of our e-triage tool for improving patient care and reducing associated costs. However, substantial refinement utilising larger datasets and robust machine learning algorithms is required.

## Appendix

### Sample of e-triage tool questions:

- How old are you?
- Are you male, female or would you rather not say?
- Do you currently have any problems with your knee?
- Is your knee problem worse in your left or right knee?
- Did your knee problems start after an injury? *(If yes* → *further questions on injury, if no* → *skip ‘injury’ questions)*

Example ‘injury’ questions:

- When you injured your knee, was it a twisting/pivoting injury?
- When you injured your knee, did you experience a sudden deceleration or stopping force?
- When you injured your knee, did you experience a blow to the upper aspect of your shin
- When you injured your knee, did you experience a tearing sensation
- When you injured your knee, did you experience a popping sensation
- Were you able to weight bear immediately after the injury occurred?
- Did your knee swell up within 2 hours of your injury?
- When you injured yourself, was it on poor ground conditions?
- When you injured yourself, did you fall onto a bent knee
- When you injured yourself, did your knee bend too far backwards?
- Did you injure your knee in a high speed/energy accident (e.g. car accident or fall from height)?

- Do you have pain in your knee? (If yes → further questions on pain, if no → skip ‘pain’ questions)
- Is your knee swollen?
  - Is your knee very swollen or mildly swollen?
- Do you experience locking of your knee?
- Does your knee ever feel like it is catching?
- Does your knee ever feel like it is going to give way?
- Does your knee click?
- Do you ever get a cracking or popping sensation in your knee?
- Have you been less active recently due to your knee problem?
- Have you previously had surgery on either of your knees?
- Do you have a history of previous knee problems?
- Have you previously injured your anterior cruciate ligament?
- Do your knees ever feel stiff *(If yes → further questions on stiffness, if no → skip ‘stiffness’ questions)*
- Do you feel like your leg muscles have become weaker?
- Do you feel like you have a limited range of movement in your knee?
- Are you typically very flexible?
- Do you find it difficult going down inclines?
- Do you find it difficult walking on flat ground?
- Have you ever dislocated your knee? *(If yes → further questions on dislocation, if no → skip ‘dislocation’ questions)*
- Are you able to fully straighten your knee?
- Would you consider yourself a high-level athlete?
- Is your job very physically demanding?
- Do you do any sports that are physically demanding?
- Do you do any sports that require sudden changes of direction?
- Do you have a history of knee osteoarthritis in your close family?
- Do you find it difficult going from sitting to standing?
- Do you find it difficult to perform twisting activities or sudden changes or direction?
- Do you find it difficult to control your knee movements?
- Have you noticed that you have become increasingly knock-kneed or bow-legged?
- How tall are you (in metres)?
- How much do you weigh (in kilograms)?
